# Challenges in the diagnosis of heart failure with preserved ejection fraction in individuals with obesity

**DOI:** 10.1186/s12933-025-02612-z

**Published:** 2025-02-07

**Authors:** Bas M. van Dalen, Jie Fen Chin, Praveen A. Motiram, Anneke Hendrix, Mireille E. Emans, Jasper J. Brugts, B. Daan Westenbrink, Rudolf A. de Boer

**Affiliations:** 1 Thorax Center, Department of Cardiology, Cardiovascular Institute, Erasmus MC, Rotterdam, The Netherlands; 2 Department of Cardiology, Franciscus Gasthuis & Vlietland, Kleiweg 500, Rotterdam, 3045 PM The Netherlands; 3 Department of Cardiology, Ikazia Ziekenhuis, Rotterdam, The Netherlands; 4 Department of Cardiology, University Medical Center Groningen, University of Groningen, Groningen, The Netherlands

**Keywords:** Obesity, Heart failure with preserved ejection fraction, Diagnosis, Echocardiography, Natriuretic peptides

## Abstract

The rising prevalence of obesity and its association with heart failure with preserved ejection fraction (HFpEF) highlight an urgent need for a diagnostic approach tailored to this population. Diagnosing HFpEF is hampered by the lack of a single non-invasive diagnostic criterion. While this makes a firm diagnosis of HFpEF already notoriously difficult in the general population, it is even more challenging in individuals with obesity. The challenges stem from a range of factors, including the use of body mass index as a conceptually suboptimal indicator of health risks associated with increased body mass, symptom overlap between HFpEF and obesity, limitations in physical examination, difficulties in electrocardiographic and echocardiographic evaluation, and reduced diagnostic sensitivity of natriuretic peptides in individuals with obesity. In this review, we examine these diagnostic challenges and propose a diagnostic algorithm specifically tailored to improve the accuracy and reliability of HFpEF diagnosis in this growing patient demographic.

**Graphical abstract:**

Proposed obesity-adjusted HFpEF score. HFpEF, Heart failure with preserved ejection fraction. Created with BioRender.com.

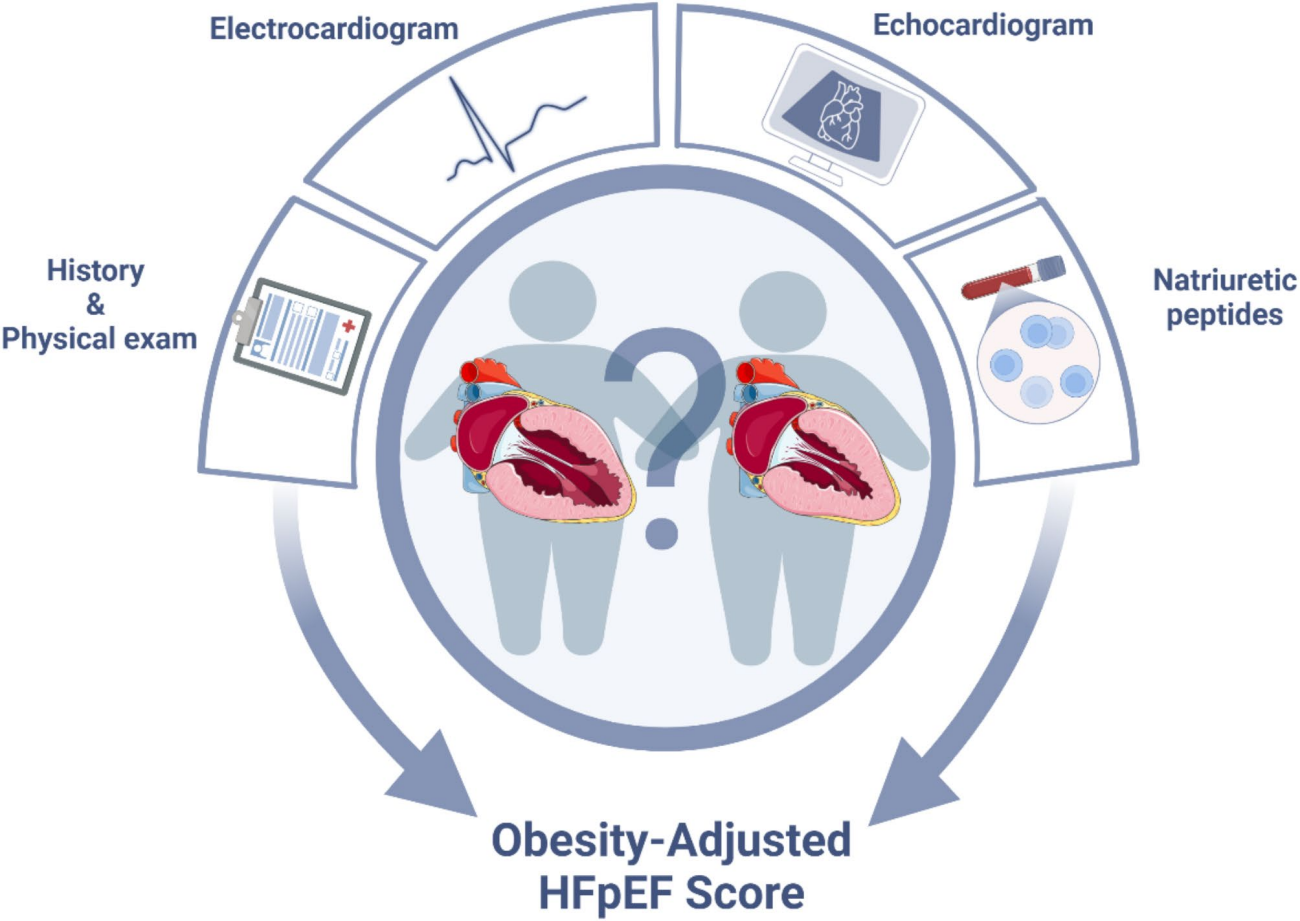

## Introduction

Obesity is a global pandemic, affecting over 650 million adults worldwide, and, if the current trend persists, it is anticipated that up to 20% of the world’s adult population (1.2 billion adults) will have obesity by 2030 [1]. Obesity, particularly when associated with increased visceral fat, has deleterious effects on the cardiovascular system and is strongly tied to development heart failure (HF), especially HF with preserved ejection fraction (HFpEF) [2–6]. A one-unit increase in body mass index (BMI) is associated with a 34% increased relative risk to develop HFpEF, and more than 80% of HFpEF patients are either overweight or obese [7, 8].

Several recommendation papers and guidelines [9–11] on the diagnosis of HFpEF have been published. Most have focused on pathophysiological mechanisms, differential response to treatment in patients with obesity, but also on obesity as a confounder in the interpretation of diagnostic tests. Diagnosing HFpEF is hampered by the lack of a single non-invasive diagnostic criterion. While this makes a firm diagnosis of HFpEF already notoriously difficult in the general population, it is even more challenging in individuals with obesity. This complexity arises due to symptom overlap, physical examination challenges, electro- and echocardiographic limitations, and reduced sensitivity of natriuretic peptides. Given the ever-increasing number of patients suffering from obesity and HFpEF, we will discuss in this review the diagnostic challenges and propose a diagnostic algorithm tailored specifically for the diagnosis of HFpEF in individuals with obesity.

## Performance of BMI as a diagnostic hallmark of obesity as compared to other anthropometric measures

Accurately identifying those with overweight and the highest risk of disease based on anthropometric parameters is complex. The most widely used method for obesity screening is the measurement of BMI [12], where obesity is defined as a BMI ≥ 30 kg/m^2^ (class 1 obesity: BMI 30–34.9 kg/m^2^; class 2 BMI 35–39.9 kg/m^2^; class 3 BMI ≥ 40 kg/m^2^). However, BMI is a non-specific indicator of body mass, as it does not differentiate between fat mass, muscle mass, and bone mass [13]. Also, the relationship between obesity and mortality risk remains controversial: a large body of evidence supports a “J-shaped” or a “U-shaped” association of body BMI with all-cause mortality [14, 15]. This discrepancy in association of BMI with mortality risk may arise, at least in part, due to the inability of BMI to differentiate between fat mass and lean body mass [16, 17]. Additionally, the relation between BMI and percentage of body fat is not linear and differs for men and women [13]. Therefore, alternative screening measures such as waist circumference (WC) and waist-to-hip ratio (WHR), which better capture abdominal fat distribution, have been incorporated into risk prediction models [18]. Over the past decade, more precise anthropometric indicators of adiposity, such as relative fat mass (RFM) [19, 20], body shape index [21] body roundness index [22] and weight-adjusted waist index [23] have been developed. Recently, it was shown that among novel and established anthropometric measures of adiposity, the relative fat mass (RFM), which is calculated from WC and height, was the strongest predictor of HF risk in the general population [24]. Future studies should determine whether such novel measures may replace the widely used measurement of BMI as the parameter of choice in diagnosing obesity.

## History and physical examination focused on heart failure in individuals with obesity

Typical HF symptoms include breathlessness, reduced exercise tolerance, and signs of fluid retention such as weight gain and ankle swelling. Individuals with obesity often experience a number of physical complaints related to excess body weight, which are largely similar to those of HF patients. This overlap can make it challenging to distinguish whether symptoms are due to HFpEF or the effects of obesity alone.

### How do the symptoms of HF and obesity overlap?

The prevalence of dyspnea on exertion in otherwise healthy individuals with obesity has been reported to be more than 40% [25]. Compared to their non-obese counterparts, individuals with obesity are nearly three times more likely to experience shortness of breath while walking uphill [26]. Functional residual capacity and expiratory reserve volume are known to be reduced in individuals with obesity, while inspiratory capacity is increased, thus maintaining a relatively normal total lung capacity [27]. The reduction in functional residual capacity increases the prevalence and severity of expiratory flow limitation [28, 29]. In addition, the excess fat around the chest wall decreases the total respiratory system compliance, leading to an increased oxygen cost of breathing [30, 31]. This results in the shallow and rapid breathing pattern typical of individuals with obesity [25]. Weight loss has been shown to ameliorate most of these pathological changes in lung volumes and oxygen cost of breathing [32], providing a causal link between obesity and these alterations.

Apart from the decreased lung function, reduced exercise tolerance in individuals with obesity can stem from several other non-cardiac causes. Musculoskeletal problems, such as osteoarthritis and joint pain, can limit physical activity due to discomfort and reduced mobility [33]. While the prevalence of osteoarthritis increases with age in the general population and is estimated to affect 40% of people over the age of 70 years, even 50–70% of individuals with obesity may experience osteoarthritis, particularly in weight-bearing joints like the knees and hips [34, 35]. Associated metabolic issues, like type 2 diabetes, can lead to fatigue and muscle weakness [36]. Additionally, increased body mass raises energy expenditure during movement, causing the “typical” HF complaint of fatigue [37]. Furthermore, increased prevalence of obstructive sleep apnea could also be related to decreased exercise tolerance in individuals with obesity [38]. Psychological factors, such as depression and low self-esteem, can also reduce motivation and physical activity levels [39]. The prevalence of depression in individuals with obesity is significantly higher than in the general population. Estimates indicate that about 30–50% of people with obesity exhibit symptoms of depression, while in the general population, the prevalence is typically around 10% [40]. This increased prevalence can be attributed to various factors, including the psychosocial impact of obesity, such as stigma and social isolation, as well as the biological effects of obesity on the brain. Lastly, deconditioning from a sedentary lifestyle decreases overall physical fitness, further limiting exercise capacity.

The overlap between obesity and HFpEF not only complicates the distinction between symptoms of each condition but also intensifies the overall symptom burden. While dyspnea is a hallmark of HFpEF, patients with obesity tend to experience more severe breathlessness and greater exertional intolerance, likely due to increased body mass, decreased chest wall compliance, and elevated respiratory workload [41, 42]. Also, obesity is associated with increased fatigue and greater limitations in daily physical activities. HFpEF patients with obesity often have a higher degree of physical disability as measured by 6-minute walk tests or similar assessments [43]. Finally, HFpEF patients with obesity generally report poorer quality of life compared to non-obese HFpEF patients, with worse scores on health-related questionnaires [44]. This may be driven by the combined effect of obesity on physical activity, mobility, and increased symptom burden.

### How do the signs of HF and obesity overlap?

A crude estimate of approximately 15.000 patients with severe obesity attending a US clinic showed that almost 75% of them had chronic leg edema [45]. There can be either local, systemic or medication-related causes. In individuals with obesity, venous insufficiency is a well-known non-cardiac cause of edema. Excess weight increases pressure on leg veins, causing fluid to leak into the surrounding tissues. Lymphedema can also occur due to impaired lymphatic drainage from adipose tissue accumulation [46]. Additionally, chronic inflammation associated with obesity can increase capillary permeability, further promoting edema. Also, relatively often used medications in these subjects, such as corticosteroids and certain antihypertensives, can induce fluid retention. Finally, commonly associated comorbidities may also promote edema. Kidney disease can lead to fluid retention and edema due to impaired filtration. Obstructive sleep apnea, common in obesity, may contribute to edema through repeated nighttime hypoxia and resultant fluid shifts [47].

While increased abdominal girth in obesity is caused by visceral fat, in HFpEF patients it may also be attributable to ascites as an expression of decompensated heart failure. Obviously, obesity tends to develop more gradually, with a consistent increase in weight over time. In the case of rapid increase in abdominal girth, this is more likely to be caused by fluid retention than fat accumulation. Shifting dullness and a fluid wave may be clinical signs of ascites. Shifting dullness is determined by percussing the abdomen while the patient changes position, revealing a shift in fluid, whereas a fluid wave is detected by tapping one side of the abdomen and feeling for a transmitted wave on the opposite side [48]. Although both phenomena are absent in abdomens with increased girth due to adiposity, it can be challenging to accurately determine this in clinical practice. Ultrasound is the most accurate non-invasive tool for distinguishing ascites from fat. It can clearly identify the presence of free fluid in the abdominal cavity, even in small amounts, making it the gold standard for diagnosing ascites [49].

Rales are a typical sign of pulmonary congestion or fluid overload. However, in patients with obesity, pulmonary congestion can occur without overt or audible crackles because the sound transmission may be dampened by the excess adipose tissue. Thus, HFpEF patients with obesity may have fewer audible crackles despite significant fluid overload. A similar principle applies to heart murmurs. Significant valve stenoses or insufficiencies may not be heard due to the insulating effect of fat tissue. Imaging tools, such as a chest X-ray or echocardiogram may be needed to provide objective evidence of the presence or absence of pulmonary congestion or other lung pathology, and valvular abnormalities.

The primary techniques of physical examination—inspection, palpation, auscultation, and percussion—are essential for physicians to assess normal physiology and detect pathology. However, these methods are compromised when viscera and vasculature are surrounded by a (thick) layer of adipose tissue, which may severely limit the quality of the physical exam [50, 51]. Despite the widespread obesity epidemic, many medical students and residents receive no formal training on adapting the physical examination for patients with obesity. It is crucial to emphasize the need for physicians to overcome these challenges to ensure optimal care for these patients [52].

## The electrocardiogram in obesity

Obesity is linked to numerous electrocardiographic (ECG) irregularities. While some of these are harmless, others may indicate changes in heart structure related to obesity and its associated conditions.

Obesity is associated with a leftward shift in P wave, QRS and T wave axes that is directly related to the severity of obesity and is reversible with substantial weight loss [53]. The PR-interval, QRS duration and the ST-segment are considered to be relatively unaffected by obesity [53]. In case of severe obesity, T wave flattening in the inferior and lateral leads is commonly observed. This is thought to occur due to the leftward and horizontal displacement of the base of the heart caused by excessive abdominal fat [54, 55]. Growing evidence suggests that obesity, especially central obesity, is linked to delayed ventricular repolarization, indicated by a prolonged corrected QT interval [56–58].

**Fig. 1:**
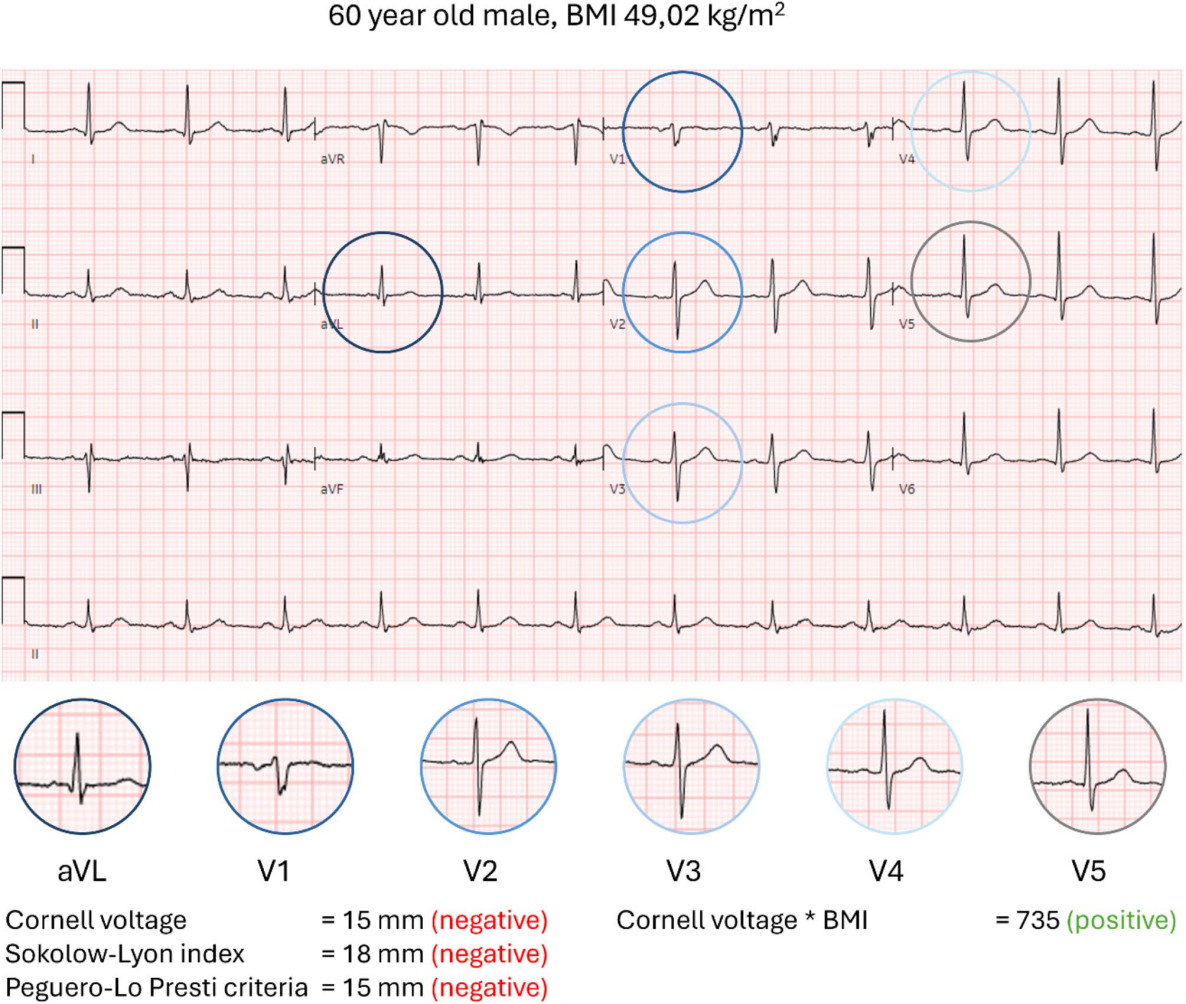
Adjusted Cornell criteria for left ventricular hypertrophy on electrocardiogram. Electrocardiogram of a 60-year-old male patient with obesity class 3 that meets the criteria for left ventricular hypertrophy based on the adjusted Cornell voltage*BMI, (RaVL + SV3)*BMI ≥ 700 mm*kg/m^2^. The diagnosis of left ventricular hypertrophy was confirmed by an echocardiogram. Note that none of the other criteria were positive. BMI, body mass index.

Left ventricular hypertrophy (LVH) is common in people with obesity, even without hypertension [59, 60], and is linked to a higher risk of cardiovascular disease and death [61, 62]. ECG criteria for diagnosing LVH have been in use since 1914 [63]. Nowadays, the two most commonly used ECG criteria are the Cornell voltage [64] and the Sokolow–Lyon index [65]. Despite a generally high specificity, most ECG criteria for LVH lack sensitivity [66]. The value of these criteria is particularly questionable in individuals with obesity because obesity is responsible for geometrical and electrophysiological changes of the heart and ECG voltages may be attenuated by subcutaneous adipose tissue [67–70]. Therefore, adjusted criteria have been proposed. Angeli et al. [71] introduced a correction to the Cornell voltage by BMI to improve the performance of ECG criteria for LVH in individuals with obesity (Fig. 1). However, in a recent study of our group it was shown that while the sensitivity of such a criterion increased as compared to the conventional criteria, sensitivity was still only 53%, and this increase was on the cost of a decrease of specificity to 72% [72].

## The echocardiogram in obesity

Diagnosis of HFpEF requires objective demonstration of congestion. Current guidelines recommend using several echocardiographic parameters to help guide the diagnosis of HFpEF [9, 10]. However, challenges arise when performing echocardiography in individuals with obesity, which can be categorized into two primary issues.

Firstly, poor image quality is a primary concern in echocardiography for patients with obesity. Excess adipose tissue can impede ultrasound waves, leading to suboptimal visualization of cardiac structures. This limitation can hinder accurate assessment of cardiac function and structure. Consistent with other studies [73, 74], our group found that image quality is generally better in normal-weight individuals compared to those with severe obesity. However, contrary to earlier retrospective studies, we observed that the feasibility and reproducibility of cardiac function and structural parameters remain comparable when the echocardiogram is performed as part of a scientific study, by a technician not hindered by limited time [75]. Because of the technical challenges in individuals with obesity, physicians should be allowed to use more time, and preferably all the time necessary, to identify the optimal windows. The widespread idea of echocardiography usually being non-diagnostic in patients with obesity may lead to a self-fulfilling prophecy; poor image quality during the acquisition of images may be more easily accepted by the operator. However, when the technician is given sufficient time to make all necessary technical adjustments for optimal image quality, the majority of key parameters can be accurately measured.

Secondly, reference values and cutoff points derived from lean populations may not be directly applicable to individuals with obesity, necessitating the use of obesity-specific reference ranges and interpretation criteria. The external mechanical constraint on the heart due to obesity, particularly from abundance of epicardial adipose tissue within the pericardial sac, is believed to cause an uncoupling between cardiac wall stress and intracardiac pressures [76]. Recent data have revealed that circulatory congestion is underestimated by echo-Doppler indicators such as the ratio of the peak early LV filling velocity and early diastolic mitral annular velocity (E/e′ ratio) in patients with obesity [76]. Also, normalization of parameters to body surface area (BSA) does not work for individuals with obesity. For instance, the current European Society of Cardiology (ESC) guidelines recommend indexing left atrial volume (LAV) to BSA [77]. However, since BSA is mainly driven by an increase in fat mass, indexing LAV to BSA can lead to overcorrection of LAV among patients with obesity, potentially normalizing LA dilatation. Additionally, LAV indexed to BSA is an isometric measure that assumes a linear relationship between LAV and BSA, which is incorrect because heart and body size do not grow proportionally [78]. Alternatively, it has been suggested that a more appropriate measure to define LA enlargement in patients with obesity could be to use allometric scaling by indexing LAV to body height squared [79, 80]. Also, it is recommended to index LV mass (LVM) by exponentiating body height by 2.7 in individuals with obesity [81].

**Fig. 2:**
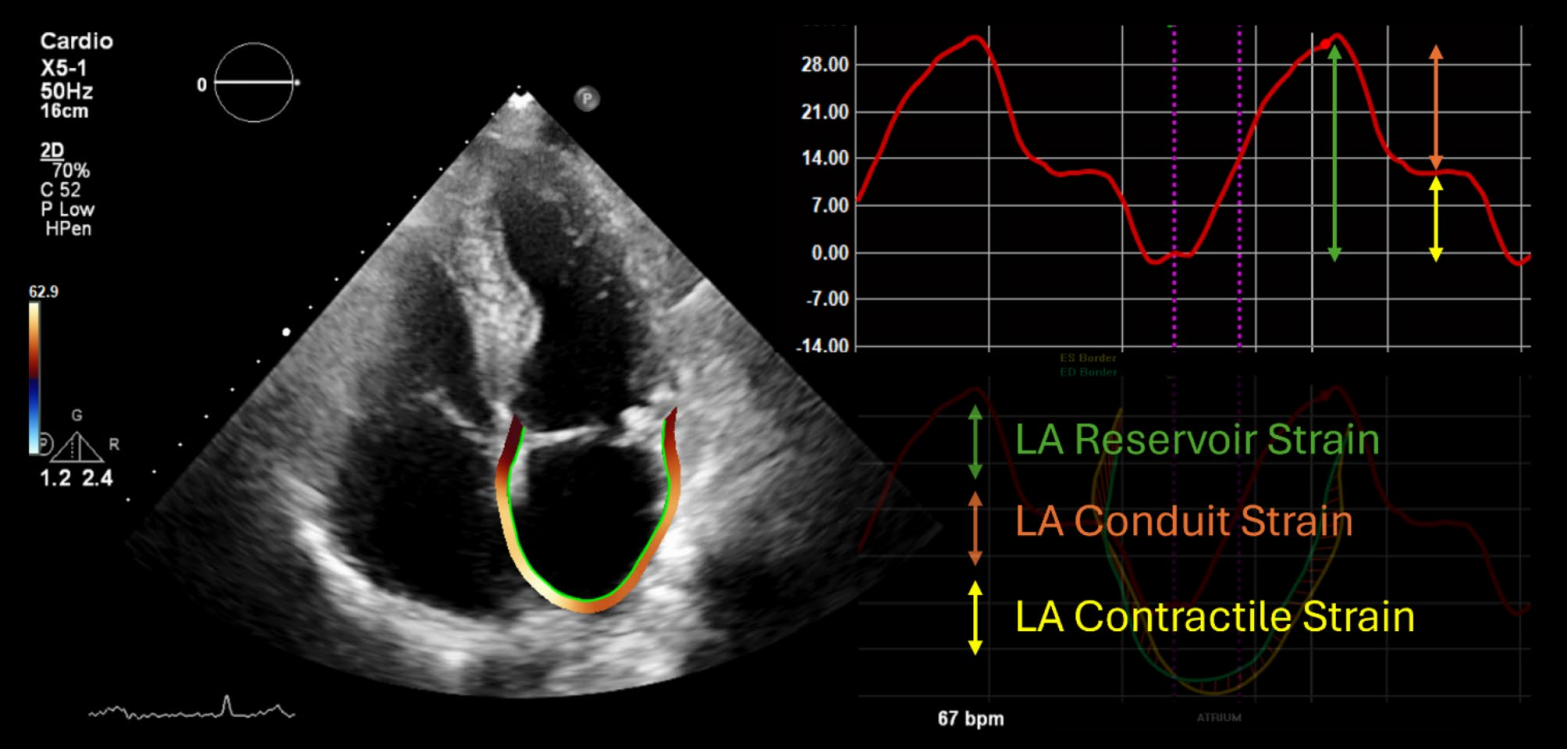
Measurement of left atrial strain. LA function based on the three phases of the LA cycle: LA reservoir strain, LA conduit strain, and LA contractile strain. LA, Left atrial

The estimation of LA pressure using the E/e’ ratio can be challenging in individuals with or without obesity due to an unavailable or unreliable E-wave caused by heart rhythm abnormalities and/or mitral valve disease [82]. In such individuals, assessing LA strain potentially carries added clinical and prognostic value (Fig. 2) [83, 84]. In patients with normal EF, LA reservoir strain < 18% has high specificity for increased filling pressures [85]. Furthermore, in a recent study it was shown that in individuals with obesity, impairment of LA strain occurs before diastolic dysfunction assessed by conventional echocardiographic parameters may become apparent [86]. Therefore, assessment of LA strain (Fig. 2) could have important added value in identifying patients at higher risk of obesity-related HFpEF.

By acknowledging and addressing these challenges, we may improve the accuracy of echocardiographic assessments in individuals with obesity, leading to better diagnosis and management of HFpEF in this population.

## Natriuretic peptides in obesity

The natriuretic peptides (NPs), B-type natriuretic peptide (BNP) and N-terminal pro B-type natriuretic peptide (NT-proBNP), are essential biomarkers used in diagnosing HF and are incorporated into the ESC guidelines for HFpEF diagnosis [9]. However, it is crucial to interpret NP levels within the clinical context, as in acute setting NP levels are higher than in chronic setting. Normal NP levels also do not exclude HFpEF. Notably, up to 20% of a population of patients with a mean BMI of 34 kg/m^2^ and invasively confirmed HFpEF, have NPs below diagnostic thresholds [87]. Moreover, in individuals with obesity, several challenges affect the accuracy and reliability of NP measurements, complicating their use for HFpEF diagnosis.

It is well-established that circulating NP levels are reduced in individuals with obesity, both with and without HF [88].The underlying mechanisms remain incompletely understood. Given the different mechanism of clearance of different types of NPs, the reduction is most likely to be the result of lower release of NPs [89]. LVH, commonly present in obesity [59, 60], can normalize LV end-diastolic wall stress, thereby reducing NP release. Additionally, external mechanical constraints on the myocardium, probably due to the abundance of epicardial adipose tissue within the pericardial sac, may contribute to this normalization of wall stress. Consequently, NP levels in individuals with obesity may not accurately reflect LV filling pressure, leading to underdiagnosis of HFpEF. Furthermore, NP deficiency in obesity has significant sex- and age-associated components [90]. The inverse relationship between NT-proBNP and obesity is more pronounced in females than males. Additionally, sex modifies the direct relationship between NT-proBNP and age, with age-related increases in NT-proBNP being more substantial in males compared with females. These findings suggest that NT-proBNP levels in individuals with obesity should be interpreted with consideration of both sex and age.

Several studies have evaluated the diagnostic accuracy of NPs for diagnosing HF in populations with obesity in acute settings. Lowering the NP cut-off levels seems to maintain diagnostic sensitivity for HF in patients with obesity [88]. A recent study tested adjusting NT-proBNP cut-offs based on BMI to improve acute heart failure (AHF) diagnosis. In a cohort of 2038 patients, 25% were obese, with 53% of them diagnosed with AHF. The diagnostic accuracy of NT-proBNP was lower in patients with obesity. Adjusting the cut-offs—by 33% for BMI 30–34.9 kg/m and 50% for BMI ≥ 35 kg/m^2^—improved sensitivity from 96.7 to 98.2%, resulting in reduced missed AHF cases. Specificity, however, decreased slightly, from 84.9 to 76.5% [91]. A prospective cohort study reported decreased sensitivity of both BNP and NT-proBNP in diagnosing decompensated HF in individuals with increased BMI [92]. Using established cut-off points, BNP sensitivity dropped to 85% in overweight individuals (BMI 25–30 kg/m^2^) and 81% in individuals with obesity (BMI > 30 kg/m^2^). NT-proBNP sensitivity decreased to 68% and 69% in overweight and obesity, respectively. In this study it was also found that the diagnostic accuracy of BNP was comparable to NT-proBNP across all BMI categories. Although not yet implemented in the current ESC guidelines for HFpEF diagnosis, the Heart Failure Association of the ESC suggests that lowering the established cut-off concentrations by up to 50% may optimize diagnostic accuracy. Therefore, a very low BNP cut-off concentration of < 50 pg/mL is recommended to rule out HF in patients with obesity in the acute setting [11].

While it is recommended to reduce the NP cut-off value by 50% to improve the accuracy of HF diagnosis in patients with obesity, this adjustment is based predominantly on data from acute-care settings. Most available data on NP thresholds for chronic HF are derived from studies involving symptomatic patients referred by general practitioners [93, 94]. To date, however, no clinical trial has evaluated the diagnostic accuracy and utility of NP measurement specifically for patients with obesity in the outpatient, chronic HF contexts. Given the established observation that NP levels are lower in obesity in acute HF scenarios, it is reasonable to hypothesize that similar pathophysiological patterns would hold in the chronic setting.

In summary, while NPs remain valuable biomarkers for HF diagnosis, their interpretation in individuals with obesity is less reliable and differential cutpoints may be considered to maintain diagnostic accuracy. However, even with these adjustments, challenges remain in accurately diagnosing HFpEF in this population.

## Proposed diagnostic criteria for HFpEF in individuals with obesity

HFpEF diagnosis comes with important prognostic information and might lead to further investigation to identify the underlying etiology and to initiate appropriate treatment. However, a conclusive diagnosis of HFpEF remains challenging in subjects with or without obesity. Two relatively novel scoring systems have been devised to attain a more accurate HFpEF diagnosis. The HFA-PEFF scoring system incorporates a pretest evaluation of risk, echocardiography and NP testing, functional testing, and determination of the final etiology [9]. The H2FPEF score takes several factors into account, including obesity, the use of 2 or more antihypertensive medications, atrial fibrillation, ageing, and echocardiographic signs of pulmonary hypertension or elevated filling pressures [10]. Both scoring systems yield results on a continuous scale, categorizing the likelihood of HFpEF as low, intermediate, or high. Patients deemed to have an intermediate likelihood require further invasive hemodynamic assessments, which are technically complex, costly, and carry inherent risks.

In the H2FPEF score, comorbidities, such as obesity and atrial fibrillation, are included as risk enhancers, and a high score is diagnostic for HFpEF. However, this means that if a patient with a high H2FPEF score would lose weight below the threshold, or undergoes effective ablation of atrial fibrillation, the diagnosis HFpEF can effectively be dismissed. As such, the H2FPEF score largely considers HFpEF to be a myocardial manifestation of the cardiac milieu, rather than a primary myocardial disease per se.

The HFA-PEFF score can be determined when results of an initial clinical and demographic history in individuals presenting with symptoms or signs compatible with HF, are suggestive of HFpEF. Since there is no single non-invasive diagnostic criterion for HFpEF, a combination of echocardiographic measurements of cardiac structure and function, and NP levels is recommended. However, as highlighted in this review, cut-off values of NPs and echocardiographic parameters commonly used for diagnosing HFpEF are often inadequate for individuals with obesity.

**Fig. 3:**
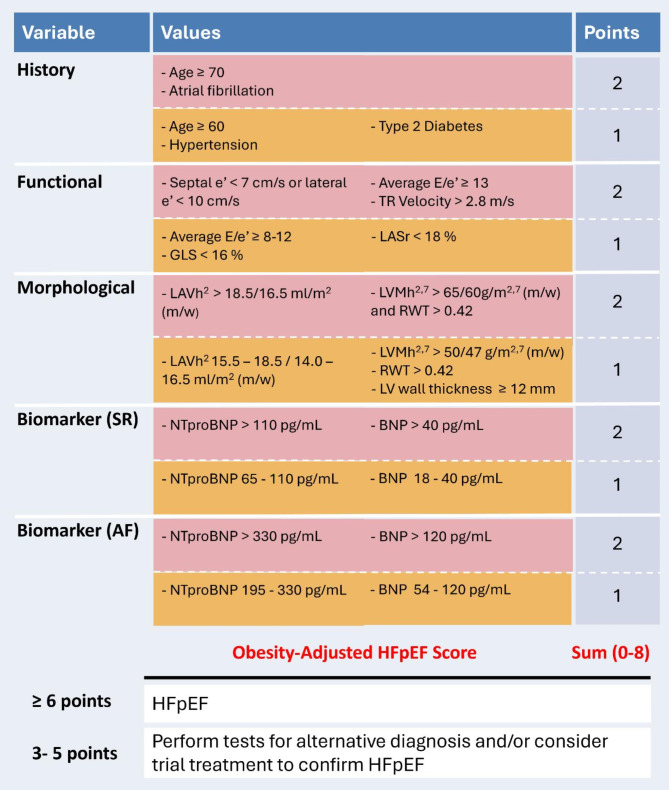
Hypothetical scheme for an obesity-adjusted HFpEF score in individuals with BMI ≥ 30 kg/m^2^. Similar to the HFA-PEFF scoring system, each domain can provide up to 2 points: 2 points are awarded if any major criterion in that domain is met, or 1 point if no major criteria are met but at least one minor criterion is fulfilled. Even if multiple major criteria are satisfied within the same domain, the maximum contribution from that domain remains 2 points. Similarly, if multiple minor criteria are met without any major ones, the domain still only contributes 1 point. Points are not cumulative within the same domain and are only combined when they come from different domains. **Disclaimer** This is not a validated score, but a hypothetical scheme to provoke thinking about the diagnosis of HFpEF in obesity. E/e’, early diastolic transmitral flow velocity/early diastolic mitral annular velocity; TR, tricuspid regurgitation; GLS, global longitudinal strain; LASr, left atrial strain reservoir phase; LAVh^2^, left atrial volume indexed by height^2^; LVMh^2.7^, left ventricular mass indexed by height^2.7^; m/w, men/women; RWT, relative wall thickness; NTproBNP, N-terminal pro B-type natriuretic peptide; BNP, B-type natriuretic peptide; AF, atrial fibrillation; HFpEF, heart failure with preserved ejection fraction.

Considering the limitations of the established criteria for diagnosing HFpEF in individuals with obesity, it would be reasonable to adjust the criteria of the HFA-PEFF diagnostic workup to ensure that the score still has sufficient value. We would like to emphasize that, while there is a relatively strong scientific base for the individual parameters used for HFpEF diagnosis, the constructed HFA-PEFF score is based on consensus among experts in the field of HFpEF and not on e.g. validation with invasive measurements or confirmed prognostic value. In line with this premise, we herein propose an obesity-adjusted HFpEF score that combines elements of the HFA-PEFF (and H2FPEF) score with specific adjustments to account for the physiological and pathophysiological impact of obesity (Graphical abstract, Fig. 3). The rationale behind the obesity-specific cut-off values for the echocardiographic parameters and NPs is described in detail and supported by references in this review, and is summarized below.

In accordance with the H2FPEF score, we believe that age and comorbidities that increase the risk of HFpEF may be considered to be included in the scoring system for individuals with obesity. Therefore, we added these criteria in a separate column as “History”. Since diabetes plays an important role in obesity-related HFpEF [95, 96], we included type 2 diabetes as a minor criterion as well. While HFpEF predominantly affects older adults, obesity can promote its development at a younger age by accelerating cardiovascular aging processes and contributing to metabolic and inflammatory pathways that drive cardiac dysfunction [4, 97]. Therefore, we propose age ≥ 60 years as a minor, and ≥ 70 years as a major criterion.

For functional echocardiography measurements, we propose using an E/e’ ratio ≥ 13 as a major and between 8 and 12 as a minor functional criterion in individuals with obesity. These cut-off values are slightly lower than the ones used in the HFA-PEFF and H2FPEF score, because, as mentioned before, in the presence of obesity pulmonary capillary wedge pressure is known to be higher for any E/e’ ratio as compared to individuals with lower BMI [76]. Furthermore, LA strain is used as a minor functional criterion as well. Regarding the morphological echocardiography measurements, given that BSA normalization is inappropriate in obesity, we recommend indexation of LAV to height^2^ and LVM to height^2.7^. Also, recognizing that NP levels are often lower in individuals with obesity and may not meet diagnostic thresholds for HFpEF [11] we propose reducing the cut-off values by 50%. This adjustment aligns with recent recommendations from the Heart Failure Association (HFA) of the ESC [11].

In the HFA-PEFF scoring system, a score of ≥ 5 points is considered diagnostic for HFpEF and in case of a score of 2–4 points further investigations are needed. Given our addition of the “History” criteria, these cut-off values should be adjusted to ≥ 6 points and between 3 and 5 points, respectively.

It is important to recognize that the sensitivity and specificity of the HFA-PEFF scoring system vary significantly depending on the chosen cut-off point [98, 99]. At a threshold of ≥ 2 points, the HFA-PEFF score demonstrates high sensitivity (99%) but low specificity (19%), resulting in a considerable number of false positives. Conversely, a threshold of ≥ 5 points achieves high specificity (93%) but at the cost of sensitivity (69%), leaving a substantial number of HFpEF cases undiagnosed. This tradeoff is particularly relevant in individuals with obesity, where the diagnostic markers are often confounded by adiposity-related factors, further complicating the identification of HFpEF. Moreover, the intermediate risk category (2–4 points) remains a diagnostic gray zone, with a substantial proportion of HFpEF patients—up to 36% in some studies—falling into this category [98]. By adjusting the criteria and/or the cut-off values of these criteria to values that better suit individuals with obesity, we aim to improve the diagnostic sensitivity at the high-risk threshold with our proposed scoring. While this may slightly reduce specificity, it is a tradeoff that we believe is justified to address the issue of underdiagnosis of HFpEF in this challenging population.

It is worth acknowledging that the further investigations advised in the HFA-PEFF scoring system in case of an intermediate score, stress exercise echocardiography or invasive hemodynamic measurements, are inherently linked to limitations in individuals with obesity. Although we have previously discussed the acceptable feasibility and reproducibility of assessing cardiac function and structural parameters by echocardiography in individuals with obesity, the effectiveness of stress echocardiography in this population remains uncertain. Also, bleeding risk of invasive procedures is known to be increased in individuals with obesity [100], making invasive hemodynamic measurements less attractive. Therefore, our approach emphasizes the importance of conducting a thorough search for alternative explanations for symptoms in patients with an intermediate obesity-adjusted HFpEF score. If anginal symptoms are suspected or there is a high-risk profile for vascular disease, further diagnostics should be performed to assess potential underlying coronary artery disease. For patients experiencing dyspnea on exertion, we recommend a chest X-ray and pulmonary function testing, or referral to a pulmonologist for further diagnostics, depending on the treating physician’s preference. For those presenting with edema, we suggest referral to a phlebologist for further evaluation. In the absence of an alternative explanation for such symptoms in a patient with an intermediate score, a trial treatment with a loop diuretic should be considered. In case of reduction of symptoms after initiation of a loop diuretic, the diagnosis of HFpEF may be made, although the potential impact of a placebo effect should be duly considered. In case of persistent diagnostic uncertainty, further analysis with invasive measurements or stress echocardiography can be considered at centers with sufficient expertise in this area.

As discussed, the two most commonly used scoring systems, H2FPEF and HFA-PEFF, have notable limitations in diagnosing HFpEF in individuals with obesity. These challenges arise from distinct clinical and pathophysiological features associated with obesity, which are not fully captured by existing criteria. Recently, emerging techniques such as machine learning, combined with new insights into the mechanisms driving cardiac dysfunction in obesity, have facilitated the development of novel diagnostic scores. For example, the HFpEF-JH score proposed by Bermea et al., which incorporates predictors such as BMI, estimated glomerular filtration rate, left ventricular mass index, and the left atrial to left ventricular volume ratio, has shown promise in improving diagnostic accuracy in this patient population [101]. Further research comparing the performance of novel scoring systems and our proposed obesity-adjusted HFpEF score, within an invasively confirmed cohort of HFpEF patients with obesity, is warranted. Such comparisons would provide critical insights into the strengths and limitations of each approach, ultimately advancing our ability to diagnose and manage HFpEF in this challenging population.

## Conclusion

The diagnosis of HFpEF in individuals with obesity comes with unique and complex challenges. This narrative review has explored the multifaceted difficulties inherent in this process, from symptom overlap and the intricacies of physical examination to the limitations of the ECG, echocardiography, and NPs sensitivity. Given these challenges, HFpEF may be underdiagnosed in this population when following current guidelines. To address this issue, we have proposed an obesity-adjusted HFpEF score, a modified diagnostic algorithm tailored specifically for individuals with obesity. Continued research and refinement of criteria are essential to further improve diagnostic accuracy and ultimately improve patient outcomes.

## Data Availability

No datasets were generated or analysed during the current study.
